# Neuronal injury biomarkers GFAP and neurofilament light chains (NfL) are associated with neurotoxicity and endothelial dysfunction in adult patients treated with antiCD19 CART cells

**DOI:** 10.1007/s10238-026-02051-4

**Published:** 2026-01-19

**Authors:** Eugenio Galli, Anna Modoni, Luca Battistini, Monica Rossi, Gisella Guerrera, Ilaria Pansini, Alessandro Corrente, Stefan Hohaus, Patrizia Chiusolo, Federica Sorà, Paolo Calabresi, Simona Sica

**Affiliations:** 1 Dipartimento di Scienze di Laboratorio ed Ematologiche, Fondazione Policlinico Universitario Agostino Gemelli IRCCS, Rome, Italy; 2 Sezione di Ematologia, Dipartimento di Scienze Radiologiche ed Ematologiche, Università Cattolica del Sacro Cuore, Rome, Italy; 3 Dipartimento di Neuroscienze, Organi di Senso e Torace, Fondazione Policlinico Universitario Agostino Gemelli IRCCS, Rome, Italy; 4 Unità di Neuroimmunologia, IRCCS Fondazione Santa Lucia, Rome, Italy; 5 Dipartimento di Neuroscienze, Università Cattolica del Sacro Cuore, Rome, Italy

**Keywords:** Chimeric antigen receptor t-cells (CAR-T), Neuronal damage, Neurofilament (NfL), GFAP, ICANS

## Abstract

Chimeric antigen receptor T-cell (CAR-T) therapies targeting CD19 have revolutionized the treatment of B-cell malignancies, but their use is frequently complicated by immune effector cell-associated neurotoxicity syndrome (ICANS). The underlying mechanisms include endothelial dysfunction, blood–brain barrier disruption, and neuroinflammation. Circulating biomarkers of neuronal and astroglial injury, such as neurofilament light chain (NfL) and glial fibrillary acidic protein (GFAP), may provide insight into ICANS pathophysiology and serve as predictive tools. We conducted a retrospective study of 34 adult patients treated with anti-CD19 CAR-T cells for B-cell malignancies. Serum NfL and GFAP were measured at infusion (day 0) and day 7 using ultrasensitive immunoassays. Baseline GFAP and NfL levels correlated with endothelial activation markers, including mEASIX and lactate dehydrogenase, but not with demographic variables. ICANS of any grade occurred in 34% of patients, and baseline levels of both GFAP and NfL were significantly associated with ICANS development and with steroid requirement. In contrast, changes in biomarker concentrations between day 0 and day 7 were not statistically significant overall, although individual variability was observed. At day 7, elevated NfL levels correlated with laboratory evidence of disseminated intravascular coagulation (DIC), prolonged clotting time, and C-reactive protein elevation. GFAP elevation was also linked to coagulopathy, particularly prolonged clotting time. Baseline serum levels of GFAP and NfL predict the risk of ICANS and the need for corticosteroid intervention in CAR-T-treated adults, supporting their role as biomarkers of pre-existing neuronal susceptibility. Furthermore, their association with coagulation abnormalities underscores the interplay between neurotoxicity, endothelial stress, and systemic inflammation. These findings highlight the potential clinical utility of integrating GFAP and NfL into multimodal biomarker panels to improve risk stratification and management of CAR-T neurotoxicity.

## Introduction

Chimeric Antigen Receptor T-cell (CAR-T) therapies have shown remarkable efficacy in treating various hematological malignancies, offering new hope for patients with otherwise refractory diseases [1]. However, the increasing use of CAR-T therapy has raised concerns regarding associated neurotoxicity. Immune effector cell-associated neurotoxicity syndrome (ICANS) is the most common neurological toxicity observed after CAR-T therapy, with a reported incidence of approximately 30–50%, depending on the clinical setting and product used [2]. Clinically, ICANS presents as a “frontal encephalopathy,” typically manifesting with a variety of symptoms including language disturbances, confusion, spatial and temporal disorientation, focal neurological deficits, epileptic seizures, and, in severe cases, encephalopathy with cerebral edema [3]. Given that ICANS is one of the most feared complications of CAR-T therapy, current research focuses on identifying clinical and biochemical predictive markers to enable careful patient selection, closer monitoring, and timely management of side effects. The pathophysiology of ICANS involves a complex interplay of immune dysregulation, cytokine storm, blood-brain barrier (BBB) disruption, neuroinflammation, and neuronal dysfunction [4]. A critical aspect of CAR-T-related neurotoxicity is the BBB, an extremely selective interface between the bloodstream and the brain that regulates the transport of chemicals and cells into brain tissue. BBB damage may result from CAR-T therapy through several mechanisms, including T-cell activation, pro-inflammatory cytokine production, and recruitment of other immune cells, leading to increased BBB permeability [5]. This disruption allows potentially toxic substances to enter the brain, triggering a cascade of neuroinflammatory events. The endothelium of cerebral blood vessels constitutes the main structural component of the BBB. Endothelial damage may occur directly through CAR-T interactions with receptors on endothelial cells or indirectly via the release of inflammatory mediators. During active cytokine release syndrome (CRS), biomarkers of endothelial impairment and predisposition to disseminated intravascular coagulation—such as increased levels of factor VIII (FVIII), von Willebrand factor, prolonged PTT, and reduced platelet and antithrombin levels—can be observed in peripheral blood [5]. The Endothelial Activation and Stress Index (EASIX) has emerged as a potential predictive marker for CAR-T-related neurotoxicity. EASIX is calculated using platelet count, creatinine, and lactate dehydrogenase (LDH) levels. Elevated EASIX levels have been associated with an increased risk of neurotoxicity in patients undergoing CAR-T therapy. A modified EASIX (mEASIX), which replaces creatinine with C-reactive protein, has been proposed to predict both acute toxicities—CRS and ICANS—as well as disease outcomes, and has been correlated with subclinical coagulopathy [6, 7].

Neurofilaments are key cytoskeletal components of neurons, providing structural stability through a fibrillary network. They are also involved in axonal transport in the central and peripheral nervous systems and in dendritic branching, where they help stabilize N-methyl-D-aspartate (NMDA) receptors within the neuronal plasma membrane [8]. The most abundant neurofilament in serum and cerebrospinal fluid (CSF) is the light chain neurofilament (NfL), a 68 kDa intermediate filament that has been validated as a fluid biomarker for neuronal damage in various neurological conditions [9]. Astrocytes play a fundamental role in the central nervous system, including structural support, BBB maintenance, and response to injury. Astrocytes can also act as a major source of complement components, cytokines, and chemokines in response to stimuli. Astrocyte activation correlates with neuroinflammation and is associated with increased levels of glial fibrillary acidic protein (GFAP) [10, 11].

Over the past five years, several studies have explored the potential correlation between CAR-T neurotoxicity and biomarkers indicative of BBB damage, neuronal injury, or astrocyte activation, in both pediatric and adult populations. In adult retrospective studies, baseline elevations in serum NfL were reported in two cohorts, preceding lymphodepletion and correlating with ICANS severity [12, 13]. NfL levels remained elevated for up to 30 days after infusion, suggesting that pre-existing neuronal damage may predispose patients to ICANS via microvascular injury and BBB breakdown [13]. A more recent single-center retrospective study of 150 adult patients treated with CAR-T cells found that elevated serum NfL at the time of leukapheresis could serve as an early predictor of ICANS. Additionally, higher serum NfL levels at CAR-T infusion correlated significantly with ICANS severity [14].

Finally, a recent case-control study of 159 adult patients with refractory high-grade B-cell lymphomas found that baseline serum NfL levels did not stratify ICANS risk, likely due to prior chemotherapy or bridging therapy. Interestingly, increases in serum NfL seven days post-infusion positively correlated with ICANS grade 2 or higher, preceding the onset of clinical symptoms [15].

## Aim

In this paper we present a retrospective study focusing on blood–brain barrier and neuronal damage biomarkers during ICANS in patients treated with anti-CD19 CAR-T cells, in order to identify a useful biomarker of ICANS and to clarify some pathophysiologic aspects of this form of neurotoxicity.

We aimed to answer three questions:

1. Are baseline levels of NfL and GFAP associated with any baseline biological or clinical parameter?
2. Do NfL and GFAP levels change from baseline after CAR-T infusion?
3. Is pre-existing or ongoing neuronal damage associated with neurotoxicity and coagulopathy?

### Methods

Inclusion criteria: adult patients (aged 18 years and above) affected by B-cell malignancies, specifically diffuse large B-cell lymphoma (DLBCL), other large B-cell lymphomas, and acute lymphoblastic leukemia (B-ALL) eligible for CAR-T treatment between January 2021 and January 2023 were consecutively enrolled in this study. Exclusion criteria: patients affected by pre-existing neurological conditions and/or other severe psychiatric or cardiovascular disorders were excluded. All patients underwent a brain MRI before CAR-T infusion. All participants provided informed consent for the anonymized use of their data. The present study was performed in agreement with the Declaration of Helsinki and was approved by the local Ethics Committee (ID 4879 Prot 0020777/22 Amendment 09/2024).

Clinical and biological characteristics of patients were collected from medical records, and serum samples were collected on the day of CAR-T infusion (day 0) and seven +/- 3 days after infusion.

Risk of toxicities after CAR-T infusion was stratified with the CAR-HEMATOTOX and the mEASIX, which combine inflammatory and hematological parameters as described in the literature [6, 7, 16].

Disseminated intravascular coagulation (DIC) was identified with the score proposed by the International Society on Thrombosis and Haemostasis (ISTH): in that system, platelet count, fibrinogen, PT, and fibrin-related markers are graded 0-to-3, and a score of 5 or more defines a condition of active DIC [17].

Eight ml samples of peripheral blood collected from each patient were centrifuged within 3 h from sampling for 15 min at 3000 revolutions per minute (rpm) for serum separation, which was immediately stored at -80 °C. Frozen serum samples were then carried on dry ice to the Neuroimmunology Lab, Santa Lucia Foundation IRCCS, Rome (IT) for quantitative determination of NfL and GFAP. NfL and GFAP concentrations were quantified using the fully automated ultrasensitive Single Molecule Array (SiMoA) SR-X Analyzer (Quanterix Corporation, Massachusetts). Measurements were performed with commercially available NF-Light™ Advantage Kit (item #104073) for NfL and the GFAP Discovery Kit (item #102336) for GFAP, following the manufacturer’s instructions and a two-step digital immunoassay protocol [18]. Briefly, frozen serum samples were stored at − 80 °C until analysis and thawed at room temperature immediately prior to testing. Samples were centrifuged at 10,000 × g for 5 min to remove debris. All calibrators (neat) and serum samples were measured in duplicate, according to the manufacturers’ instructions, including appropriate standards and internal controls. Serum samples were diluted 1:4 using the provided sample diluent prior to loading into individual wells; this dilution factor was validated for the accurate quantification of both NfL and GFAP. To minimize intra-assay variability, all longitudinal samples from each subject were analyzed within the same run. Assay sensitivity parameters were defined according to the manufacturer’s specifications. For the NfL assay, the analytical lower limit of quantification (LLOQ) was 0.640 pg/mL, with a limit of detection (LOD) of 0.141 pg/mL. For the GFAP assay, the LLOQ was 1.37 pg/mL, the LOD was 0.26 pg/mL, and the assay demonstrated a dynamic range up to 4000 pg/mL. Assay precision was assessed based on performance characteristics provided in the kit datasheets. The intra-assay coefficient of variation (CV) was reported to be < 8% for NfL and < 10% for GFAP, while the inter-assay CV was < 12% for NfL and < 15% for sGFAP across the relevant concentration ranges.

While comparing baseline variables we adopted tools from descriptive statistics, such as the equal-variances T-test for continuous variables or Chi-square test. Similarly, the ISTH score and other continuous variables were tested with the T-test. Associations between GFAP/NfL levels and continuous parameters, mostly concerning coagulation or inflammation, were calculated with Spearman’s correlation. The predictive value of baseline GFAP/NfL for ICANS and steroid administration was assessed with logistic regression analysis. All analyses were conducted with NCSS 2020 (NCSS, LLC, Kaysville, Utah, USA, ncss.com/software/ncss).

The study was conducted in accordance with the principles of the Declaration of Helsinki and approved by the local ethics committee (ID 4879, Prot. 0020777/22, Amendment 09/2024). Written informed consent was obtained from all participants for the anonymous use and publication of their data.

## Results

Thirty-nine patients were considered for inclusion, and five were excluded for not having available serum samples. Of the 34 patients, 20 were female, and the median age was 55 years (range 28–75). Baseline samples were available for all patients.

### Baseline GFAP and NfL

The median previous lines of therapy was two, with 16 (47%) patients having received a previous hematopoietic stem cell transplant. Twenty-two (64.7%) patients had received no previous lumbar punctures, while 12 patients had received 3–8 intrathecal injections of methotrexate. Other characteristics of the population are shown in Table 1.

**Table 1:** Baseline characteristics of the population

Variable		Median /Patients
Age	Median	55.5 yrs (range 28–75, IQR 18.5)
	Up to 60	21 (61.8%)
	Over 60	13 (38.2%)
Gender	Females	20 (58.8%)
	Males	14 (41.2%)
Previous lines	Median	2 (range 2–6, IQR 1)
	Up to 2	25 (73.5%)
	> 2	9 (26.5%)
Previos LP	Median	0 (range 0–8, IQR 4)
	0–1	22 (64.7%)
	> 1	12 (35.3%)
Stage	I-II	12 (35.3%)
	III-IV	22 (64.7%)
ECOG	0–1	26 (76.4%)
	> 1	8 (23.6%)
CAR-HEMATOTOX	Low risk	13 (38.3%)
	High Risk	21 (61.7%)

Legend: NfL neurofilaments light chain; GFAP glial fibrillary acidic protein; LP lumbar punctures; ECOG Easter Cooperative Oncology Group Performance Status scale; CAR-HEMATOTOX: see text

**Fig. 1:**
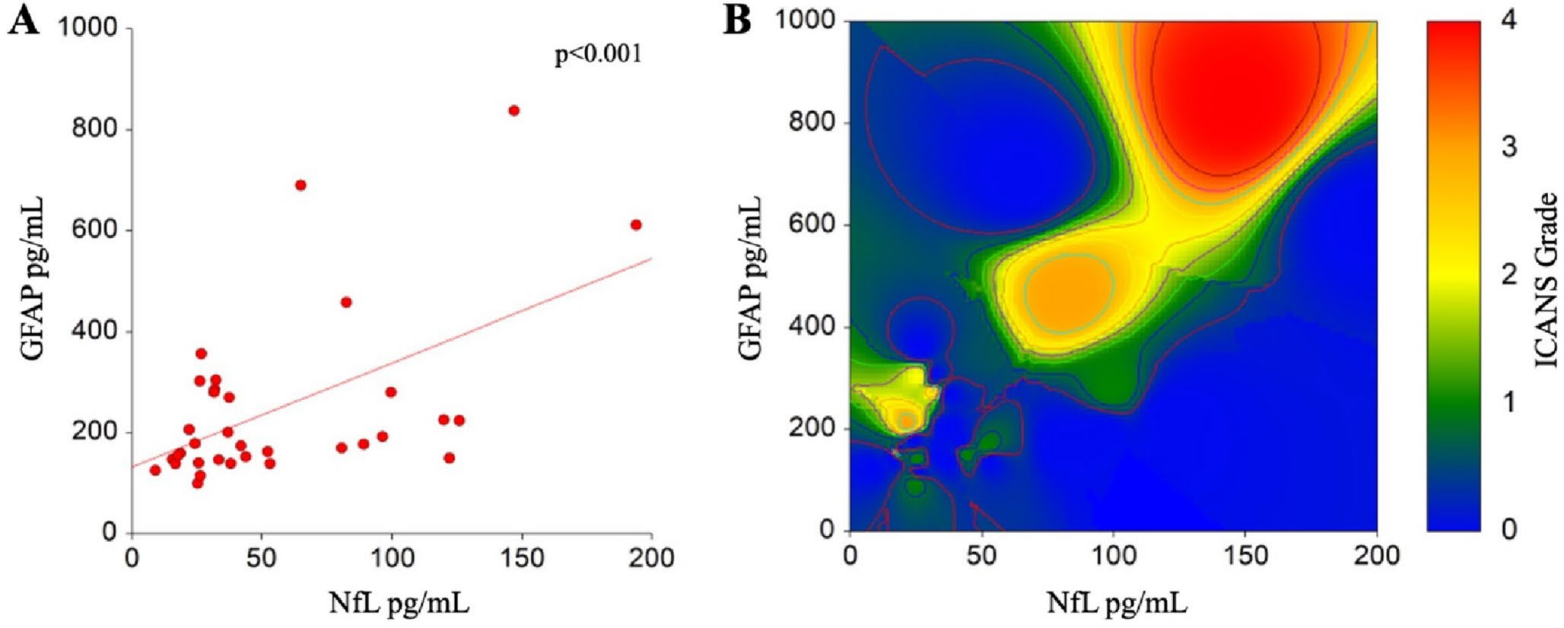
Correlation between baseline levels of GFAP, NfL, and prediction of ICANS. A- Spearman’s correlation between baseline (day 0) levels of GFAP and NfL. B- High baseline levels of GFAP (Y axis) and NfL (X axis) are predictive of the development of higher grades of ICANS, represented as an heatmap, in which more severe toxicities are represented by warmer colors

Median baseline GFAP and NfL were 178.04 pg/mL (range 99.66-837.95) and 37.11 pg/mL (range 8.97-193.98), respectively. We observed a positive correlation between baseline GFAP and NfL (Spearman’s correlation rho = 0.45, *p* < 0.001) (Fig. 1A).

**Fig. 2:**
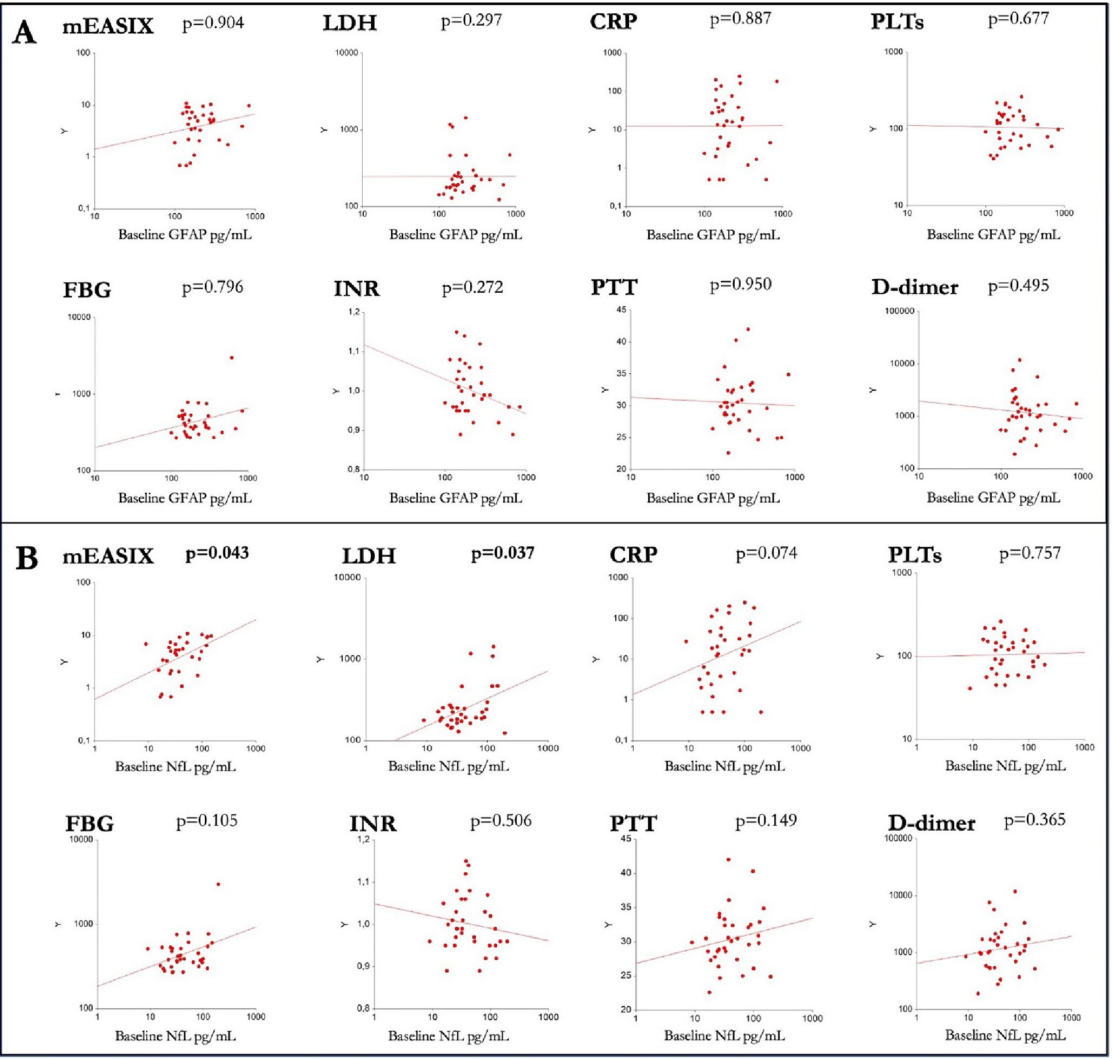
Correlations of baseline GFAP (**A**) and NfL (**B**) with inflammatory and hematological biomarkers. The tested biomarkers are represented in the Y axis in all the correlation plots, with all variables presented in a log10 scale

We tested baseline GFAP and NfL with clinical and demographic characteristics of patients, finding that patients with ECOG > 1, higher mEASIX and higher LDH at day 0 showed higher levels of NfL on the same day. No other clinical, demographic, or laboratory variable was associated with GFAP and NfL at baseline (Table 2; Fig. 2).

**Table 2:** Baseline GFAP and NfL according to subgroups of population

Variable at day 0		GFAP day 0 pg/mL (95% C.I.)	*p* value	NfL at day 0 pg/mL (95% C.I.)	*p* value
Age	Up to 60	169.9 (140.5-225.6)	0.309	41.87 (25.59–96.34)	0.368
	Over 60	201 (155-357.1)		32.24 (24.16–82.43)	
Gender	Females	183.28 (146.7-280.61)	0.881	39.89 (31.55–82.43)	0.361
	Males	178.04 (147.67-280.57)		29.15 (18.57–80.63)	
Previous lines	Up to 2	169,99 (147.67–224,06)	0.057	37,9 (31.78–80.63)	0.793
	> 2	280,61 (159,66–611,55)		26,6 (24,16–147,04)	
Previous LP	0–1	173.68 (140.46-224.06)	0.183	34,33 (25.21–53.06)	0.467
	> 1	231,17 (149.77-458.11)		39,6 (26.07–96.34)	
Stage	I-II	166.43 (146.7-286.25)	0.472	32.55 (18.57–80.63)	0.677
	III-IV	192.55 (149.77-280.57)		39.89 (25.59–82.43)	
ECOG	0–1	175.775 (147.67-269.95)	0.075	32.78 (25.21–43.72)	**0.026**
	> 1	224.83 (140.46-690.22)		92,44 (25,59–125,95)	
CAR-HEMATOTOX	Low risk	177,37 (140.46–206.4)	0.510	26,24 (17.66–89.12)	0,987
	High Risk	224.06 (149.77-286.25)		37,92 (31.55–64.94)	

Legend: NfL neurofilaments light chain; GFAP glial fibrillary acidic protein; LP lumbar punctures; ECOG Easter Cooperative Oncology Group Performance Status scale; CAR-HEMATOTOX: see text

### Impact of GFAP and NfL on the severity of ICANS

ICANS of any grade (at least G1) was experienced by 11/34 patients (34%), graded 1, 2, or 3–4 in 5 (14.7%), 2 (5.9%), and 4 (11.8%) patients, respectively. Dexamethasone administration was necessary in 10 (29.4%) patients, and in one case an admission to the Intensive Care Department was predisposed.

Baseline levels of GFAP were predictive of ICANS (Fig. 1B), with medians of 177.4 (no ICANS) vs. 206.4 (ICANS) pg/mL, and an odds ratio of 2,08 (95% CI 1.01–4.28, p value 0.044). Patients with higher baseline GFAP were more likely to receive steroids (OR 2.39, 95% CI 1.14–5.01, p value 0.020).

Similarly, baseline levels of NfL are predictive of ICANS (Fig. 1B), with an odds ratio associated with a one-unit increase in NfL of 2,08 (95% 1.01–4.24, p value 0.047). As for GFAP, also patients with higher baseline levels of NfL were more likely to receive steroids (OR 2.37, 95% CI 1.13–4.96, p value 0.021). When testing GFAP and NfL together in multivariate logistic regression, only baseline NfL remained significant in predicting ICANS (OR 0.59 95%CI 0.59–1.01 *p* = 0.595, and OR 2.06 95%CI 1.01–4.22, *p* = 0.048, respectively).

In a paired T-test comparison, median levels of GFAP did not significantly change between day 0 and day 7 (*p* = 0.588). Similarly, median levels of NfL did not change between baseline and day 7 (*p* = 0.464). Despite this, we noticed that some patients experienced an increase in levels, while in some others GFAP and/or NfL decreased.

Levels of GFAP and NfL measured at day 7 were not significantly different in patients with or without ICANS (*p* = 0.505 and *p* = 0.910, respectively), nor did having received at least one dose of tocilizumab lead to significant differences in GFAP nor NfL measured at day 7 (*p* = 0.691 and *p* = 0.490, respectively). Patients receiving tocilizumab had a higher risk of experiencing ICANS (OR 1.91, 95% CI 1.29–2.83, *p* = 0.001).

### Correlation between GFAP and NfL, and risk of coagulopathy

We explored whether neuronal damage, measured with circulating levels of GFAP and NfL, could be associated with active coagulopathy, defined by a ISTH score of 5 or more.

At day 0, only one patient (3%) had an ISTH score consistent with active DIC, possibly in concomitance with laboratory lysis syndrome after the Flu-Cy lymphodepleting regimen. On day 7, 12 (35%) patients had subclinical DIC.

Regarding neuroinflammation, median NfL at day 7, levels were higher in patients with DIC, with median values of 67.31 (95% CI 26.64-122.13) vs. 32.78 (95% CI 24.16–43.72) pg/mL for patients with or without DIC (*p* = 0.024), respectively. On the same day, patients with higher levels of NfL also had higher CRP and prolonged aPTT clotting time (*p* = 0.047 and *p* = 0.013, respectively).

Regarding astrocyte activation, median GFAP levels were 253.08 (95% CI 140.46-458.11) vs. 175.77 (95% CI 147.67–206.4) pg/mL for patients with or without DIC (*p* = 0.086) respectively. Patients with higher GFAP were more likely to experience an elevation of aPTT clotting time (*p* = 0.004).

## Discussion

To date, blood NfL and GFAP levels have been recognized as reliable biomarkers of neuronal damage across a range of neurodegenerative and neuroinflammatory disorders [19].

Moreover, blood NfL levels are regarded as clinically valuable, as they can reflect specific clinical and radiological features of several neurological conditions and may even function as prognostic indicators [20–23]. However, these biomarkers are not disease-specific [9]. NfL is a structural protein found in the axons of both central and peripheral neurons and its elevation can reflect axonal damage originating from either the CNS or the peripheral nervous system (PNS) (as in vincristine-mediated damage), while GFAP is a highly specific biomarker for central astroglial injury or activation. Therefore, the combined measurement of NfL and GFAP enhances the ability to differentiate between central and peripheral neurotoxicity, providing a more accurate assessment of the site and extent of neuronal injury in patients receiving neurotoxic chemotherapy [10, 19, 20]. Both GFAP and NfL can be affected by physiological and pathological factors, including chemotherapy [24]. Accordingly, in our study population, the baseline levels of these biomarkers are not directly comparable with median values reported for the general population [25] as our patients had undergone multiple lines of chemotherapy, including second-line agents capable of crossing the blood–brain barrier, such as cytarabine. Interestingly, baseline GFAP levels exhibited a trend toward higher values in patients who had received a greater number of therapy lines. In our population, GFAP and NfL levels did not significantly differ between baseline and day + 7 timepoints. This report does not align with the findings reported in the pediatric population studied by Gust et al., who also measured GFAP and NfL in both serum and cerebrospinal fluid. These authors observed a global increase in GFAP and NfL across all patients, regardless of their risk of developing ICANS [26]. We may hypothesize that, in the pediatric population, the detrimental effects of prior treatment regimens on the central nervous system are more pronounced due to the immaturity of cerebral tissues and the greater plasticity of neuronal functions.

Baseline NfL levels and GFAP levels predicted the development of ICANS, and patients with higher markers of baseline neuronal damage were more likely to require steroids immediately after CAR-T infusion to mitigate the neurotoxicity. Despite the correlation between GFAP and NfL, we found that only NfL levels were independent predictors of ICANS, possibly underlying a prominent role of neuronal damage in favoring neurotoxicities. We also identified a correlation between baseline NfL levels, worse ECOG, and biological markers of endothelial inflammation, as assessed by the mEASIX score and LDH, with a trend toward significance also observed for CRP. These data highlight the connection between worse clinical conditions, systemic inflammation- most likely correlated to the active burden of lymphoma (LDH)- and impairment of the endothelial structures and BBB permeability. In our opinion, this baseline condition may represent an important finding, as it supports the hypothesis that pre-existing blood–brain barrier disruption, in conjunction with endothelial dysfunction, may constitute an impacting and measurable risk factor for the development of neurotoxicity. This is in partial misalignment with what observed by Vilanseca and colleagues, but in line with what was observed by Butt and colleagues and by the French group, suggesting incorporating neurofilaments into the decision-making process to guide product selection and therapeutic decisions, to which we add enriching data on coagulation and endothelial activation [13–15]. In this sense, we observed that NfL levels measured at day 7 correlate with the occurrence active laboratory DIC as per ISTH score, prolonged clotting time (PTT) and CRP elevation, possibly reinforcing the concept that, during CAR-T–related toxicities, neuronal and coagulative impairment may share endotheliopathy as a common mechanistic pathway. Additionally, GFAP elevation was associated with a prolonged clotting time (PTT).

Patients receiving tocilizumab did experience more ICANS, as already reported by Larue M and colleagues [14], despite this observation being biased by the presence of higher CRS grades in the tocilizumab-exposed population which involve higher inflammatory burden. Notably, we did not observe higher NfL and GFAP levels at day 7 in patients receiving tocilizumab compared to the others, suggesting that this drug, or the subsequent IL-6 peak, did not trigger neuronal damage or particularly disrupt BBB permeability.

We acknowledge that the limited patient sample may raise some concern about reproducibility. As per neuronal damage markers, this study aligns with previous data, adding relevant input on endothelial damage and coagulopathy; in this sense, replication in larger longitudinal cohorts is necessary to offer translational tools to support diagnosis and stratification within precision biological frameworks.

Taken together, these data may contribute to confirm that ICANS physiopathology is a complex multifactorial mechanism including endothelial pathology, BBB breakdown, microvascular injury and neuronal damage, even before clinical symptoms’ manifestation, and provide a proof-of-concept that peripheral markers of neuroaxonal and astroglial injury can help predict ICANS. Integration into multimodal biomarker panels may enhance diagnostic precision, guide treatment planning, possibly tailor prophylactic measures, and ultimately improve outcomes for this vulnerable subgroup.

## Data Availability

Data are available upon request addressed to the corresponding authors.
